# *Bacteroides maternus* sp. nov., a novel species isolated from human faeces

**DOI:** 10.1038/s41598-025-96846-2

**Published:** 2025-04-21

**Authors:** Emilene da Silva Morais, Ghjuvan M. Grimaud, Alicja Warda, Niamh Stephens, R. Paul Ross, Catherine Stanton

**Affiliations:** 1 Department of Microbiology, University College Cork, Cork, Ireland; 2 APC Microbiome Ireland, Cork, T12 YT20 Ireland; 3 Teagasc Moorepark Food Research Centre, Fermoy, Co. Cork Ireland; 4 Conway Institute of Biomolecular and Biomedical Research, University College Dublin, Belfield, Dublin, Dublin 4 Ireland; 5 Division of Biotechnology and Applied Microbiology, Department of Process in Life Science and Engineering, Lund University, 22100 Lund, Sweden

**Keywords:** *Bacteroides maternus nov*, New species, Gut microbiome, Anaerobic bacteria, Bacteria, Bacteriology

## Abstract

A novel bacterial strain, MSB163, was isolated from the stool sample of a healthy mother, 4 weeks after giving birth via vaginal delivery. Taxonomic identification tools revealed that MSB163 belongs to the genus *Bacteroides,* but it is distinct from any currently known species. The closest related species is *Bacteroides cellulosilyticus* strain BFG- 250, with an average nucleotide identity (fastANI) of 94.51%. The genome length of MSB163 is 6,440,948 bp and the GC content 42.95%. Two plasmids were identified in the whole genome sequence. MSB163 is a Gram-negative, rod-shaped, non-motile anaerobic bacterium. The optimum growth conditions were at 37 °C, pH 7 and 0% (w/v) NaCl. The respiratory quinones were the menaquinones MK- 10 and MK- 11 and C15:0 ANTEISO was the major fatty acid. The predominant polar lipids were phosphatidylethanolamine, diphosphatidylglycerol and phospholipid. According to the taxonomic results and physiological analysis, strain MSB163 represents a novel species of the genus *Bacteroides*, for which we propose the name *Bacteroides maternus*, since the type strain was isolated from the stool sample of a mother. *B. maternus* type strain (MSB163) sequencing can be accessed under the biosample ID SAMN3953129 on NCBI. The strain was deposited on BCCM/LMG Bacteria Collection under the accession number LMG 33,374 and Leibniz Institut DSMZ GMBH under the accession number DSM 117,047.

## Introduction

The human gastrointestinal tract harbours a variety of microorganisms that play an important role in health and disease^1,2^. The gut microbiota can synthesise a variety of compounds, including vitamins, short-chain fatty acids (SCFA), amino acids and neurotransmitters, like gamma-aminobutyric acid (GABA)^3,4^. Bacteroidota and Bacillota are two of the most abundant phyla of bacteria present in the human gut^5^, and *Bacteroides*is one of the most prominent genera of the Bacteroidaceae family, accounting for a large proportion of the gut microbiome in adults^6,7^.

*Bacteroides* are a diverse and abundant group of Gram-negative, non-spore forming, anaerobic, non-motile, rod-shaped bacteria found in the human gastrointestinal tract. They are recognised for their ability to digest a large number of polysaccharides using gene clusters known as polysaccharide utilisation loci (PULs)^8,9^. *Bacteroides* species have been widely recognised as important members of the human gut microbiota, contributing to both health and disease outcomes. Lower levels of *Bacteroides *in the human gut have been implicated in disorders like obesity^10,11^, and diabetes^12^. On the other hand, some *Bacteroides *species can be opportunistic pathogens, causing clinical infections and bacteraemia^13^.

In recent years, the study of the human gut microbiome has revealed a large diversity of *Bacteroides* species, many of which have not yet been characterised. During studies of the microbiome in early life, pregnancy and puerperium, a novel *Bacteroides* species, strain MSB163, was isolated from the stool of a healthy Irish/Caucasian mother, four weeks after giving birth. The donor of the stool sample was not exposed to antibiotics and did not take probiotics from the third trimester of pregnancy until the time of sample collection. Our findings expand the known diversity of this important bacterial group by adding a novel species to the genus *Bacteroides*.

## Methods

### Ethics approval for sample collection

The Protocol and the informed consent form (ICF) have been approved by the Clinical Research Ethics Committee of the Cork Teaching Hospitals (CREC) before commencement (approval letter ECM 4 (q) 07/03/18 & ECM 3 (ppppppppp) 10/04/18). On 20 th September 2022 Protocol version 13 has been approved by the CREC (approval letter ECM 4 (q) 07/03/18 & ECM 3 (uuu) 20/09/2022). All methods were performed in accordance with the relevant guidelines and regulations approved by CREC and adhered to the principles of the Declaration of Helsinki. Informed consent was obtained from all participants and/or their legal guardians prior to inclusion in the study. Participants were provided with detailed information about the research objectives, procedures, potential risks, and benefits, and their participation was entirely voluntary.

### Isolation and ecology

Fresh faecal samples were collected and transported to the laboratory on the same day. The faecal material was serially diluted in maximum recovery diluent (MRD), spread onto pre-reduced BHIS agar plates (BHI supplemented with cysteine 0.1%(w/v), haemin 0.05% (w/v), NaHCO_3_ 0.2% (w/v), vitamin K 0.005% (w/v) and gentamicin 0.02% (w/v)) and incubated at 37 °C for 5 days in an anaerobic workstation (Ruskinn Anaerobic Chamber 10% (vol/vol) H_2_, 10% CO_2_, and 80%N_2_). Single isolates were transferred to BHIS broth, grown overnight, and stored at − 80 °C in 25% glycerol.

To identify novel strains, isolates were plated anaerobically (Ruskinn Anaerobic Chamber 10% (vol/vol) H_2_, 10% CO_2_, and 80%N_2_) in BHIS agar, grown for five days, and a pure colony was transferred to BHIS broth. The Overnight broth was used for DNA extraction (SIGMA DNA extraction kit, Germany). A PCR using the primers AllBac296f. and AllBac412r^14^ was carried out in order to identify isolates belonging to the genus *Bacteroides*. The well-known universal primers 27 F and 1492R^15^ were used to amplify the 16S rRNA region and NCBI nucleotide BLAST (rRNA database, default parameters) was used to identify isolates up to species level.

The whole genome of MSB163 was sequenced in July 2021, using Microbes NG enhanced sequencing platform (Microbes NG, UK), following Microbes NG recommended protocol. Raw short-reads from Illumina were trimmed using Trimmomatic 0.38^16^with default parameters and the sliding window parameter “SLIDINGWINDOW:4:20”, whereas long-reads from Nanopore sequencing were trimmed using NanoFilt 2.7.1^17^with default parameters and the parameter “–headcrop 75”. The quality of both short-reads and long-reads sequences were checked using fastqc 0.11.8^18^. Then, trimmed reads were assembled using the hybrid method of Spades 3.15.3 with default parameters^19,20^. CheckM 1.0.18^21^ was used to assess the quality of the assembly. We obtained a complete genome (6,440,948 bp) as well as two small contigs (8,320 bp and 4,148 bp, respectively) that we interpreted as being plasmids by identifying the presence of plasmid-specific features (i.e., the plasmid replication genes *repA* and *repB*). The plasmids and the main assembly were circularised using circlator v1.5.5 and manually inspected using a genome viewer to check for circularity. We obtained a mean coverage of 81.58X for the main assembly, while a mean coverage of 10.24X and 23.02X for the first and second plasmid, respectively.

Taxonomy was assigned up to the genus level using GTDB-tk v1.5.0^22^. To search for the closest relative species using MASH distances, the “Similar Genome Finder” of Patric v3.6.12^23^ was used. Then, a phylogenetic tree was built using the reference assemblies of all *Bacteroides *species from NCBI. Phylogeny was inferred using GToTree v1.7.07^24^ and the markers provided for *Bacteroidota*. The approximately maximum-likelihood phylogenetic tree was built with FastTree^25^. The tree was midpoint-rooted and visualised using iTol v5^26^. Additionally, 16S rRNA gene comparison between the closest related species was performed using NCBI nucleotide BLAST (16S rRNA database, default parameters). The Average Nucleotide Identity (ANI) was calculated with the closest species using fastANI 1.32^27^. Finally, the digital DNA:DNA hybridisation value was obtained between the closest related species using the dDDH calculator from TYGS^28^. The genome was annotated using Prokka v1.14^29^. Functional characterisation of protein sequences obtained from Prokka was done using eggNOG-mapper v5.0.1^30^. Synteny was assessed and visualised using the ‘pgv-mauve’ function from pyGenomeViz v0.3.1, which uses progressiveMauve for alignment.

### Characterisation and morphology

The morphology of colonies was determined after three days of growth on BHIS agar plate. Gram-staining, catalase and oxidase activity were determined by conventional methodology. Cell motility was determined on BHIS semi-solid medium (0.4% agar). *B. intestinalis* DSM 17,393 and *B. cellulosilyticus* DSM 14,838 were acquired from the DSMZ culture collection and used as reference strains in this study.

NaCl concentration range was determined by inoculating BHIS broth with added NaCl (0 to 7% w/v) and growth was assessed by measuring the OD_600_ after 24 h (WPA biowaver, USA). For the temperature test, strains were inoculated in BHIS broth at different temperatures (4, 10, 20, 28, 30, 37, 40, 44 and 55℃) and the OD_600_ was measured after 24 h (WPA biowaver, USA). For the pH tolerance test, strains were grown in BHIS broth (pH range: 4 to 10). Growth at different pH was assessed by adjusting the pH of the medium before sterilisation (4.0 to 10.0) utilising the following buffers: 100 mM sodium acetate/acetic acid and 100 mM sodium bicarbonate/sodium carbonate anhydrous.

To evaluate the ability of strains to convert glutamate to GABA, the glutamate decarboxylase activity (GAD) test was performed. The GAD test was adapted from^31^. The test solution (L-glutamic acid 0.1% (w/v), triton × 100 0.3% (v/v), NaCl 9% (w/v) and bromocresol green 0.005% (w/v)) was prepared following the method of^31^. The test strain was grown overnight in BHIS, centrifuged at 3220 × g at room temperature, washed in PBS, and resuspended in 0.5 ml of the test solution. The appearance of a blue colour after four hours was considered positive.

The ability of strains to grow in 2′-Fucosyllactose (2′-FL), an important human milk oligosaccharide, was also tested. A cell suspension of 1% (v/v) was prepared in peptone, yeast extract, and glucose broth (PYG) prepared from first principles, following the methodology from^32^, without glucose. The cell suspension (180 μl) was added to a 96-well plate with 20 μl of 10% (w/v) carbohydrate (2’-FL, lactose, glucose or cellobiose) solution. BHIS was used as positive control and PYG without carbohydrate as a negative control. Bacterial growth (OD_600_) was monitored every 10 min for 45 h using the Cerillo™ Stratus Plate Reader, at 37 ℃ in anaerobic conditions. Growth curve graphs were produced in Excel. Strains were also plated in PYG agar plates with 2’-FL as the sole carbon source and 0.04% of bromocresol purple. Bromocresol purple has a purple colour at pH above 6.8 and is yellow at pH below 5.2. A colour change from purple to bright yellow is considered positive, indicating acidification of the medium due to bacterial growth.

A series of metabolic tests were performed using Biolog plates (Biolog, USA). Biolog tests were performed in triplicate, on two different days. The maximum absorbance of each strain per carbon source was measured and can be found in the supplementary material (Table 1S). API test strips 20 A and RAPID ID 32 A (Biomerieux, France) were used following the manufacturer’s instructions. API tests were done in duplicate on at least two different occasions and the results are summarised in Tables 2S and 3S. Cellular fatty acids, respiratory quinones, polar lipids and antibiotic resistance profile analysis were carried out by DSMZ Services, Leibniz-Institute DSMZ—German Collection of Microorganisms and Cell Cultures GmbH, Braunschweig, Germany. Scanning electron microscopy (SEM) and transmission electron microscopy (TEM) analysis were performed by UCD Conway Institute of Biomolecular and Biomedical Research, University College Dublin, Dublin, Ireland.

## Results and discussion

### Genome features

**Fig. 1:**
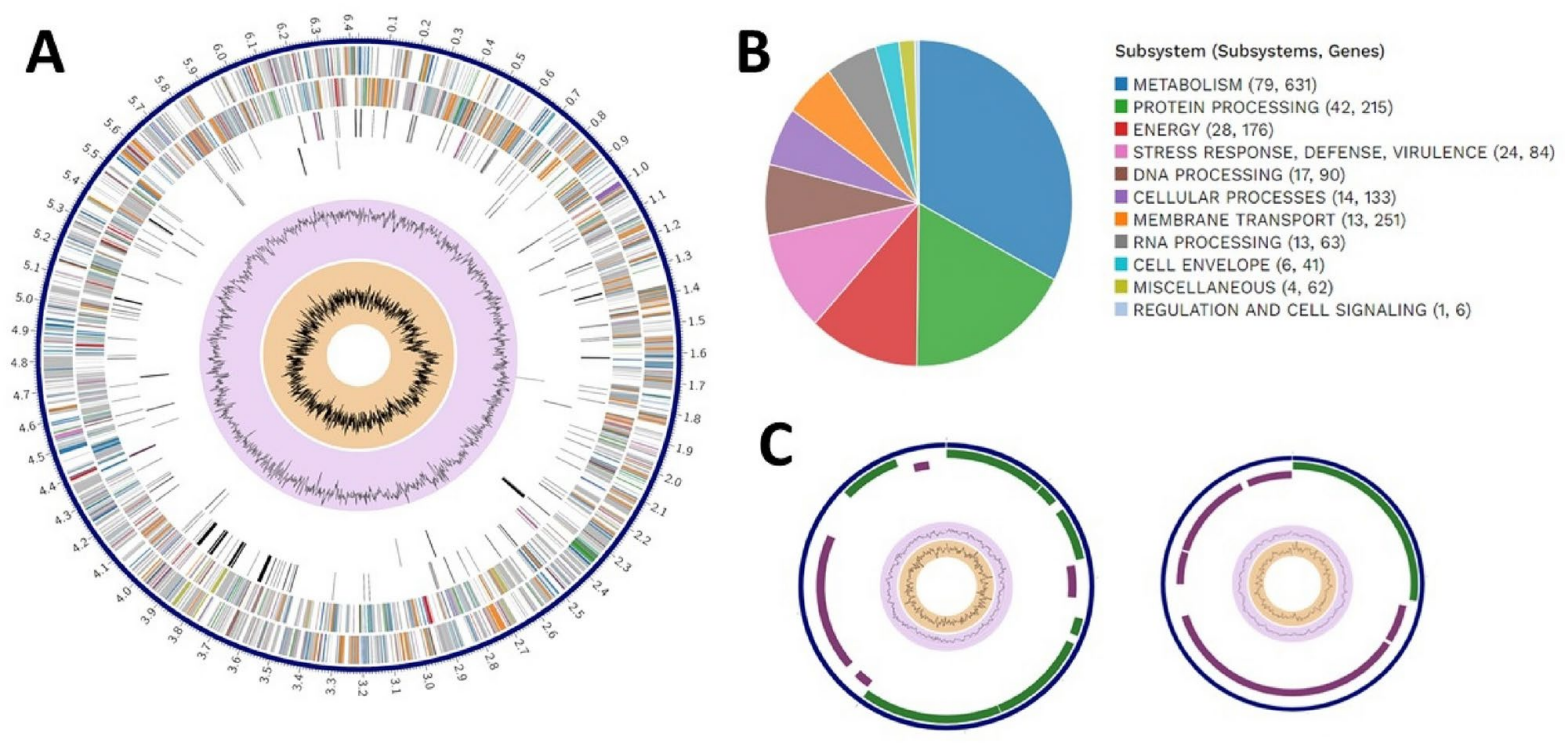
**a** Circular genome of MSB163, including GC content and GC skew. From the outer to the inner ring: CDS on the forward strand, CDS on the reverse strand, RNA genes, CDS with homology to known antimicrobial resistance genes, CDS with homology to known virulence factors, GC content and GC skew. Colours correspond to the subsystems present in b. **b** Subsystems present in MSB163 and number of genes in each subsystem. **c** Plasmid one (8,320 bp) and plasmid two (4,148 bp) circular genomes. Colours correspond to the subsystems present in b. The purple and yellow inner circles correspond to GC content and GC skew, respectively.

The GC content of strain MSB163 was determined to be 42.95% (calculated from the genome) and the genome length 6,440,948 bp. The whole genome assembly produced one contig corresponding to MSB163 genome and two small contigs corresponding to two plasmids (Fig. 1). We further analysed the genome sequence of strain MSB163 and produced a circular map including the GC content and GC skew (Fig. 1).

**Fig. 2:**
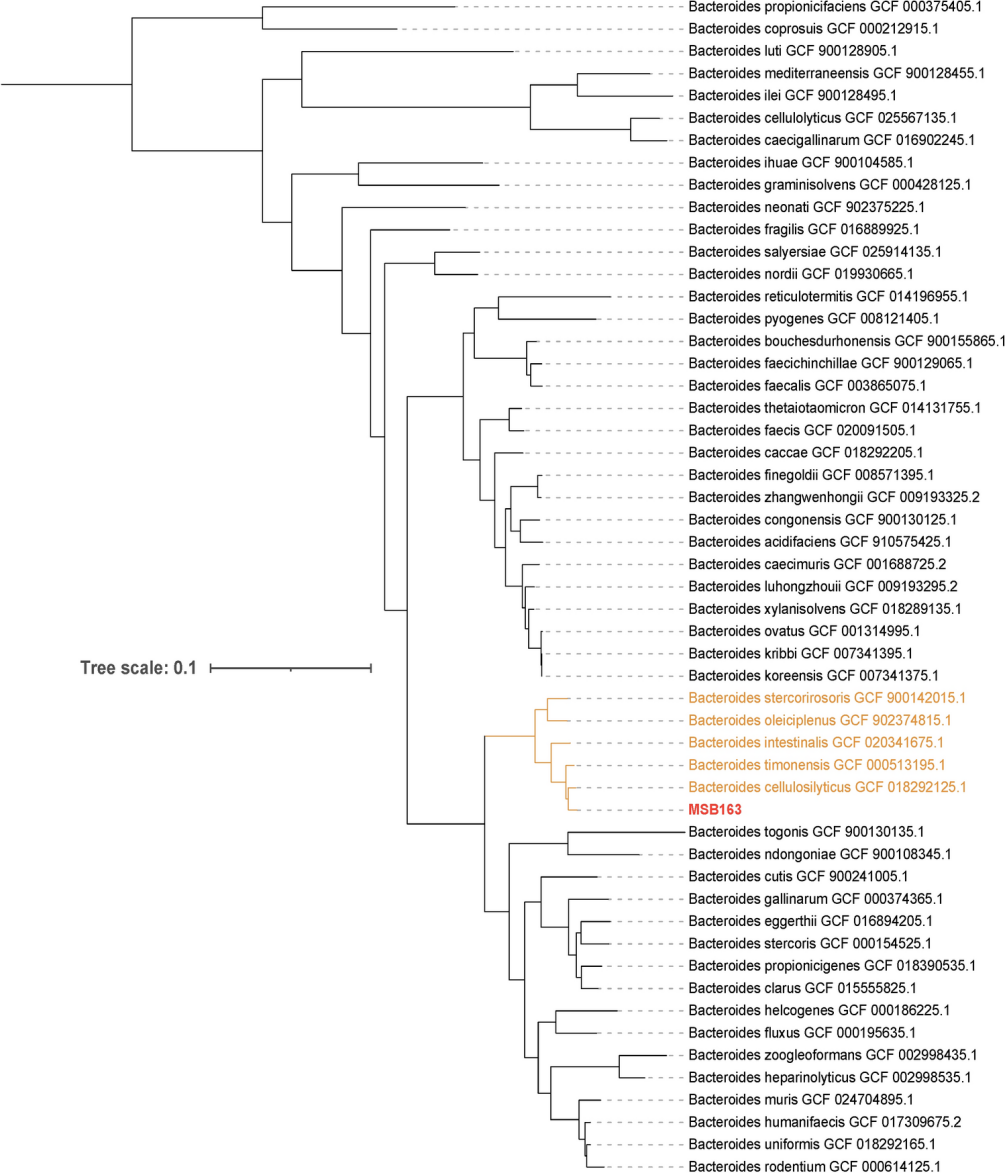
Phylogenetic tree (generated using the Maximum Likelihood method implemented in FastTree) based on whole genome sequences including all *Bacteroides* assemblies available on NCBI. Phylogeny was inferred using gene markers provided for *Bacteroidota* from GToTree. The tree shows that *B. celluloslyticus* is its closest relative to MSB163.

The completeness level of the assembly was 99.25% and the contamination level was 0.12% according to CheckM. Summarised information on the assembly of strain MSB163 can be found in Table 4S. Taxonomic identification tools, including GTDB and 16S rRNA gene blast, identified MSB163 as belonging to the genus *Bacteroides* but distinct from any currently known species. Whole genome sequences and metagenome-assembled genomes (MAGs) available on NCBI and PATRIC were investigated to identify the closest related species. MAG GCF018374845.1 (NCBI RefSeq assembly) was closely related to MSB163, with a MASH distance < 0.0162, but this MAG was unidentified. Coincidentally, this MAG was also reconstructed from a maternal stool sample. The next closest relative was *Bacteroides cellulosilyticus* strain BFG- 250, with a MASH distance > 0.0446, indicating that they are not the same species. The average nucleotide identity between MSB163 and *B. cellulosilyticus* was 94.51% (OAT OrthoANI, species threshold = 95%) and the DNA-DNA hybridisation estimated 53.80% (Genome-to-Genome Distance Calculator, species threshold = 70%), corroborating the hypothesis of MSB163 being a member of a new species. The phylogenetic tree based on the 16S rRNA gene from *Bacteroides* species available on NBCI (Fig. 1S) identified *B. oleiciplenus* and *B. stercorirosoris* as the closest relative to *B. maternus*. However, a more reliable phylogenetic tree was inferred using gene markers provided for Bacteroidota from GToTree, incorporating all *Bacteroides* species assemblies available on NCBI (Fig. 2). This analysis showed *B. cellulosilyticus* as the closest relative to *B. maternus*. *B. timonensis* is shown as the second closest species to MSB163. However, this species had only one isolate described on NCBI, with low-quality assembly data. Additionally, *B. timonensis* and MSB163 exhibited notably low synteny (Fig. 2S), indicating limited similarity in their gene order and organisation. Therefore, we decided to use *B. intestinalis* as the second comparison species, since it is well-described. *B. intestinalis* and *B. maternus* had an average nucleotide identity of 89.1%. Surprisingly, the synteny between *B. maternus* and *B. intestinalis *GCA_020341675, the type strain of the genus, was highly conserved (Fig. 2S), suggesting that the genomic architecture and order of genes on chromosomes remained relatively unchanged. No bacteriocins were identified in the genome using Bagel4^33^. In the two plasmids identified in MSB163, most features were associated with protein and DNA processing. Remarkably, plasmid one has a clindamycin resistance transfer factor, two glycosyl transferase and one glycosyl hydrolase gene (Table 4S and 5S). However, the antibiotic resistance analysis showed that a zone of 18–20 mm was produced around a disk with 10 µg of clindamycin, indicating the strain is sensitive to this antibiotic, despite the fact that it has a resistance gene on a plasmid.

### Morphology and physiology

**Fig. 3:**
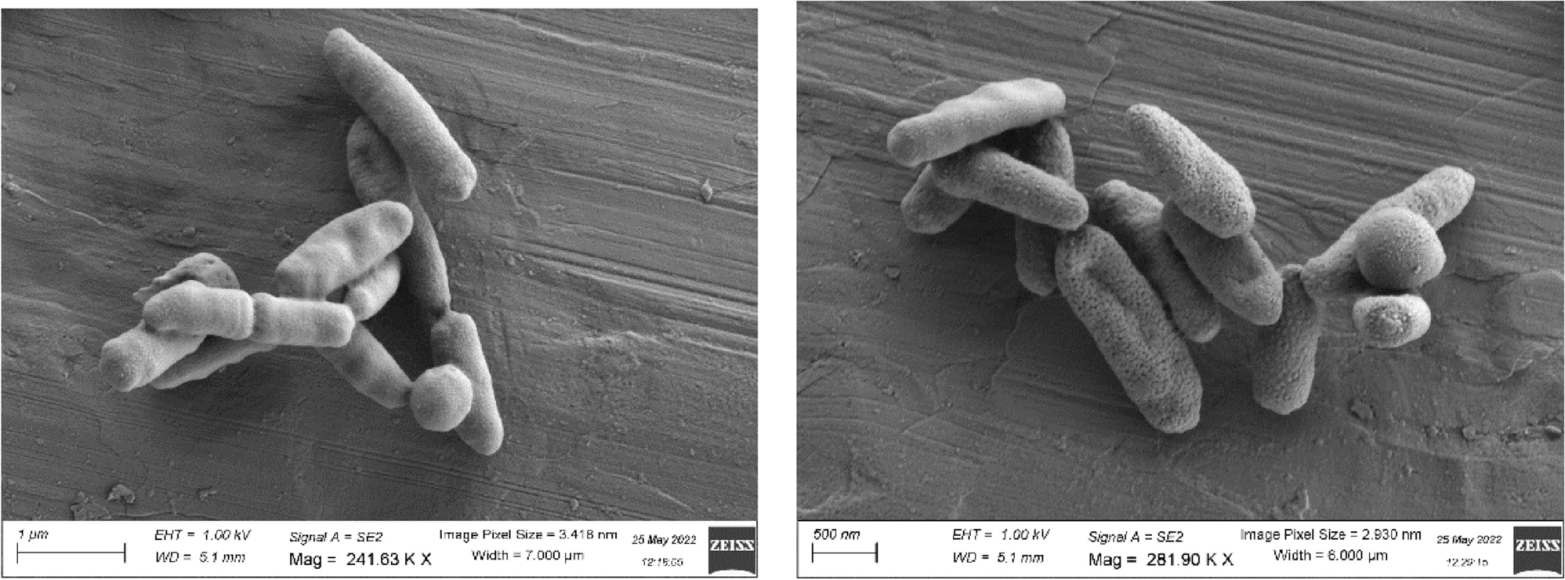
Electron Microscopy images of strain MSB163.

MSB163 colonies were 1–2 mm in diameter, circular, raised and had an entire and regular margin, with a whitish aspect. MSB163 was a gram negative, non-motile, anaerobic, catalase-positive and oxidase-negative bacterium. Electron and transmission electron microscopy (Fig. 3, Fig. 2S and Fig. 3S) were utilised to determine MSB163 cell morphology. Cells were rod-shaped and their size varied from 1.6 μm to 2.4 μm. Figure 5S shows *B. maternus* under the microscope. MSB163 grew from 28 to 40 °C, with 37 °C being the optimum temperature (Fig. 6S). The optimal salt concentration for MSB163 growth was zero, with good growth observed from 0–1.5% (w/v) of NaCl (Fig. 7S). MSB163 grew within the pH range of 6–8.5 and the optimum pH was 7. A summary of the morphological and physiological characteristics of MSB163, *B. cellulosilyticus* and *B. intestinalis* can be found in Table 1.

**Table 1:** Characteristics of MSB163, *B. cellulosilyticus* and *B. intestinalis*.

Characteristics	MSB163	*B. cellulosilyticus*	*B. intestinalis*
Shape	Rod	Rod	Rod
Gram stain	Negative	Negative	Negative
Catalase	Positive	Positive	Positive
2’-FL utilisation	No	–	–
GABA production	Yes	–	–
Growth in broth	24 h	24 h	24 h
Cell motility	No	No	No
pH	6.0–8.5	6.0–8.5	6.0–8.5
Temperature (℃)	28–40	28–40	28–40
NaCl tolerance (%)	0–1.5	0–1.5	0–1.5

The GAD test was positive, showing that MSB163 can convert glutamate to GABA. Growth in 2’-FL was negative (Fig. 8S). The catalase test showed that *B. maternus* and *B. cellulosilyticus* were catalase positive. This was unexpected, as *B. cellulosilyticus* was initially characterised as catalase negative^34^. A review of the whole genome sequence of *B. cellulosilyticus* DSM 14,838 confirmed the presence of the catalase gene. Catalase activity in *Bacteroides *species is often influenced by the type of medium utilised, with hemin concentration playing an important role^35^. Additionally, the availability of nutrients can alter the expression of catalase enzymes^36^, which explains the different results observed in our study.

**Fig. 4:**
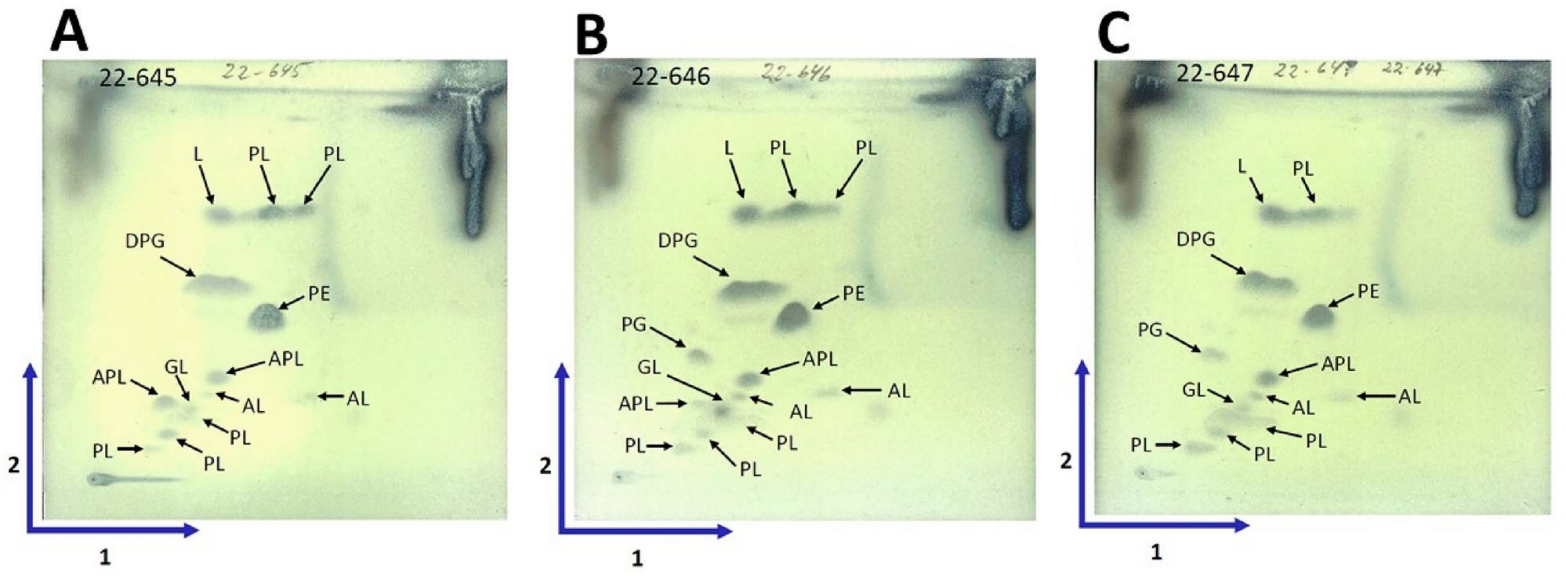
Thin-layer chromatograms of polar lipids of strains: **a** MSB163, **b** *B. cellulosilyticus* DSM 14,838 and **c** *B. intestinalis* DSM 17,393. Abbreviations: *DPG*, Diphosphatidylglycerol; *PE*, Phosphatidylethanolamine; *PG*, Phosphatidylglycerol; *APL*, Aminophospholipid; *AL*, Aminolipid; *GL*, Glycolipid; *PL*, Phospholipid.

The polar lipids test showed that phosphatidylglycerol is not found in MSB163 cell membrane, but it is present in the two reference strains, *B. cellulosilyticus* DSM 14,838 and *B. intestinalis* DSM 17,393, as shown in Fig. 4. Phosphatidylglycerol is an important component of bacterial membranes of Gram-negative bacteria, representing 20 to 25% of their membrane phospholipids^37^.

The major cellular fatty acids produced by strain MSB163 were C15:0 ANTEISO, C16:0, C17:0 ISO 3OH and C16:0 3OH. TSBA library and anaero6 and clin6 calculation methods were used. Results are summarised in Table 7S and 8S. The respiratory quinones (menaquinones) test showed that longer-chain menaquinones MK- 10 (82%) and MK- 11 (18%) were the major menaquinones found in strain MSB163 (Table 2).

**Table 2:** Respiratory quinones (menaquinones) present in MSB163, *B cellulosilyticus* DSM 14,838 and *B. intestinalis* DSM 17,393.

Strain	Menaquinones	
MSB163	MK- 10 (82%)	MK- 11 (18%)
*B. cellulosilyticus*	MK- 10 (75%)	MK- 11 (25%)
*B. intestinalis*	MK- 10 (64%)	MK- 11 (36%)

The antibiotic susceptibility test showed that no inhibition zone was observed when MSB163 was exposed to oxacillin, aztreonam, cefotaxime, ceftazidime, ciprofloxacin, ofloxacin, amikacin, gentamicin, polymyxin B, colistin sulphate, fosfomycin, kanamycin and trimethoprim-sulfamethoxazole (1:19). The biggest inhibition zones were observed when MSB163 was treated with tetracycline and linezolid, with a 20–24 mm zone. Differently from *B. cellulosolyticus* and *B. intestinalis*, MSB163 is sensitive to cefiderocol and vancomycin, with an inhibition zone of 10 mm around a disk with 30 μg of antibiotic.

For the API 20 A test, strips were incubated for 24 h, anaerobically at 37℃. MSB163, *B. cellulosilyticus* and *B. intestinalis* showed acid production from fermentation of glucose, lactose, saccharose, maltose, xylose, arabinose, esculin hydrolysis, cellobiose, mannose, raffinose and rhamnose. Interestingly, after 24 h of incubation, MSB163 had a weak activity for melezitose and sorbitol. The test was repeated with a longer incubation time, 48 h, the recommended time for slow-growing strains, and MSB163 was shown to be able to ferment melezitose and sorbitol, altering the pH of the medium, changing its colour from purple to amber, differently from *B. cellulosilyticus* and *B. intestinalis.* The longer incubation period did not affect the other test results. The full physiological and biochemical profile obtained from the API and Biolog tests can be seen in Tables 1S, 2S and 3S. On the Biolog plate assay, all three strains were negative for melezitose and sorbitol metabolism. Biolog plates were incubated on OmniLog plate reader (Biolog, USA). To minimise the impact of oxygen on strain growth, each plate was sealed in a plastic bag containing an Anaerogen anaerobic pouch (Thermo Fischer, USA), as recommended by the manufacturer, to maintain the plates as anaerobically as possible. However, this system might have affected the growth of the strains on the more challenging carbohydrate sources. In the Biolog plate assay, the strains also did not grow well in rhamnose, despite a clear positive result in the API 20 A assay. We hypothesise that these differences could be related to differences in protocol and oxygen exposure. Both tests are qualitative only.

## Conclusions

In this study, we report a new bacterial species, *Bacteroides maternus*, isolated from the stool sample of a healthy mother four weeks postpartum. *Bacteroides* is an important genus of the human gut microbiota. The identification of novel commensal species within this genus is of significant importance as it contributes to our understanding of the diverse microbial community that resides within the human gastrointestinal tract. The whole genome sequence (WGS) of *B. maternus* was compared with WGS and metagenome-assembled genomes (MAGs) available on NCBI and PATRIC databases. The closest identified sequence was an unidentified MAG (MAG GCF018374845.1—NCBI RefSeq assembly), which was also reconstructed from a maternal stool sample. The next closest relative identified was *Bacteroides cellulosilyticus*. *B. maternus* is gram-negative, anaerobic, rod-shaped bacteria, belonging to the genus *Bacteroides*. The main differences between MSB163 and its closest related species are the absence of phosphatidylglycerol on its cell membrane and the ability to ferment melezitose and sorbitol. There are also differences in the antibiotic susceptibility profile, with MSB163 being sensitive to cefiderocol and vancomycin, unlike *B. cellulosilyticus* and *B. intestinalis*. Analysis of MASH distance, DNA:DNA hybridisation and average nucleotide identity between *B. maternus* and *B. cellulosilyticus* supports the classification of *B. maternus* as a member of a new species. This discovery adds to the growing body of knowledge about the diversity of microbial species that inhabit the human gastrointestinal tract and highlights the importance of continued research in this area.

## Protologue

*Bacteroides maternus* (ma.ter.nus. L. adj., meaning maternal or relating to a mother).

Cells are strictly anaerobic rod-shaped (2.4 μm to 1.6 μm), gram-negative, catalase positive and oxidase negative. In BHIS, colonies were 1–2 mm in diameter, circular, raised and with an entire and regular margin, with a whitish aspect. Optimal cell growth occurred at 37 °C, pH 7.0, with NaCl concentration of zero. The predominant respiratory quinones were MK- 10 and MK- 11. The two major cellular fatty acid were C15:0 ANTEISO, C16:0, C17:0 ISO 3OH and C16:0 3OH. The polar lipids identified were diphosphatidylglycerol, phosphatidylethanolamine, aminophospholipid; aminolipid, glycolipid and phospholipid. Acid production was observed in the presence of glucose, lactose, saccharose, maltose, xylose, arabinose, esculin hydrolysis, cellobiose, mannose, raffinose, rhamnose, melezitose and sorbitol. The type strain, MSB163, was isolated from the human faeces of a healthy mother four weeks after giving birth, and its genomic DNA G + C content is 42.95 mol%. MSB163 was deposited at BCCM/LMG Bacteria Collection under the accession number LMG 33,374 and Leibniz Institut DSMZ GMBH under the accession number DSM 117,047. Sequencing data are available under the biosample ID SAMN39531295, project name PRJNA1067579 on NCBI.

## Supplementary Information

Supplementary Information.

## Data Availability

Sequencing data: Sequencing data are available under the biosample ID SAMN39531295, project name PRJNA1067579 on NCBI. B. Matermus strain availability: B. maternus was deposited at BCCM/LMG Bacteria Collection under the accession number LMG 33,374 and Leibniz Institut DSMZ GMBH under the accession number DSM 117,047.
