# Supplementary material for: Bacteroides maternus sp. nov., a novel species isolated from human faeces
Source: Sci Rep. 2025 Apr 21;15:13808. doi: 10.1038/s41598-025-96846-2 (PMC12012224; doi:10.1038/s41598-025-96846-2)
Supplement: Supplementary file 1 — Supplementary Information. [file 41598_2025_96846_MOESM1_ESM.docx]

**SUPPLEMENTARY TABLES AND FIGURES**

**Tables**

**Table 1S.** Absorbance (OD_600_) of MSB163, B cellulosilyticus and B. intestinalis on different carbon sources on Biolog AN MicroPlate.

| **Carbon source** | **MSB163** | ***B. cellulosilyticus*** | ***B. intestinalis*** |
| --- | --- | --- | --- |
| water | 0.00 | 0.00 | 0.00 |
| Dulcitol | 0.00 | 13.67 | 12.00 |
| Glycerol | 41.00 | 18.33 | 0.00 |
| α-Methyl-D- Galactoside | 14.00 | 13.67 | 1.67 |
| Turanose | 128.33 | 81.67 | 173.67 |
| D-Lactic Acid Methyl Ester | 3.67 | 0.00 | 4.33 |
| Alaninamide | 10.67 | 0.67 | 3.00 |
| L-Methionine | 7.00 | 4.33 | 12.33 |
| N-Acetyl-D- Galactosamine | 109.50 | 57.00 | 132.67 |
| i-Erythritol | 0.00 | 0.00 | 0.00 |
| D,L-α-Glycerol Phosphate | 0.00 | 0.00 | 0.00 |
| β-Methyl-D- Galactoside | 102.00 | 86.67 | 154.33 |
| Acetic Acid | 1.00 | 0.33 | 0.00 |
| D-Malic Acid | 0.00 | 0.00 | 0.00 |
| L-Alanine | 2.67 | 0.00 | 2.00 |
| L-Phenylalanine | 6.67 | 4.67 | 13.67 |
| N-Acetyl-D- Glucosamine | 127.00 | 69.33 | 136.67 |
| D-Fructose | 141.00 | 103.67 | 184.33 |
| m-Inositol | 0.00 | 0.00 | 0.00 |
| α-Methyl-D- Glucoside | 0.00 | 0.00 | 0.00 |
| Formic Acid | 0.00 | 0.00 | 2.67 |
| L-Malic Acid | 0.00 | 2.33 | 0.00 |
| L-Alanyl-L- Glutamine | 15.67 | 52.00 | 0.00 |
| L-Serine | 0.00 | 0.00 | 2.00 |
| N-Acetyl-β-D- Mannosamine | 113.75 | 43.67 | 128.00 |
| L-Fucose | 35.75 | 35.00 | 8.33 |
| α-D-Lactose | 128.75 | 93.00 | 174.67 |
| β-Methyl-D- Glucoside | 0.50 | 0.00 | 0.00 |
| Fumaric Acid | 4.00 | 4.67 | 4.00 |
| Propionic Acid | 4.00 | 0.00 | 0.00 |
| L-Alanyl-L- Histidine | 48.67 | 42.67 | 39.33 |
| L-Threonine | 0.00 | 0.00 | 0.00 |
| Adonitol | 7.50 | 4.00 | 0.00 |
| D-Galactose | 134.25 | 105.00 | 187.67 |
| Lactulose | 116.50 | 97.33 | 157.33 |
| Palatinose | 146.00 | 136.33 | 189.67 |
| Glyoxylic Acid | 0.00 | 0.00 | 1.67 |
| Pyruvic Acid | 4.33 | 1.00 | 1.67 |
| L-Alanyl-L- Threonine | 62.33 | 26.00 | 23.33 |
| L-Valine | 5.33 | 0.33 | 2.00 |
| Amygdalin | 96.75 | 88.00 | 159.67 |
| D-Galacturonic Acid | 54.00 | 40.33 | 46.00 |
| Maltose | 133.50 | 96.67 | 128.00 |
| D-Raffinose | 101.50 | 39.67 | 166.33 |
| α-Hydroxybutyric Acid | 0.00 | 2.00 | 0.00 |
| Pyruvic Acid Methyl Ester | 6.33 | 0.00 | 0.00 |
| L-Asparagine | 0.00 | 0.00 | 0.00 |
| L-Valine plus L-Aspartic Acid | 3.67 | 4.67 | 8.00 |
| D-Arabitol | 3.00 | 0.00 | 0.00 |
| Gentiobiose | 140.00 | 105.67 | 186.33 |
| Maltotriose | 134.25 | 90.33 | 167.67 |
| L-Rhamnose | 58.75 | 22.33 | 16.00 |
| β- Hydroxybutyric Acid | 0.00 | 0.00 | 0.00 |
| D-Saccharic Acid | 0.00 | 1.33 | 0.00 |
| L-Glutamic Acid | 0.00 | 0.00 | 0.00 |
| 2'-Deoxy Adenosine | 67.67 | 71.33 | 42.33 |
| Arbutin | 5.50 | 3.33 | 2.00 |
| D-Gluconic Acid | 22.75 | 0.00 | 0.00 |
| Maltotriose | 0.00 | 0.00 | 0.00 |
| Salicin | 38.75 | 31.00 | 29.00 |
| Itaconic Acid | 0.00 | 0.00 | 0.00 |
| Succinamic Acid | 3.33 | 0.00 | 0.00 |
| L-Glutamine | 0.00 | 0.00 | 1.00 |
| Inosine | 30.67 | 4.33 | 3.33 |
| D-Cellobiose | 113.50 | 84.33 | 171.00 |
| D-Glucosaminic Acid | 3.50 | 20.33 | 7.67 |
| D-Mannose | 133.50 | 89.67 | 162.33 |
| D-Sorbitol | 0.00 | 2.67 | 0.00 |
| α-Ketobutyric Acid | 17.00 | 14.67 | 1.00 |
| Succinic Acid | 0.00 | 0.00 | 0.00 |
| Glycyl-L- Aspartic Acid | 14.67 | 13.67 | 1.33 |
| Thymidine | 122.33 | 103.33 | 59.33 |
| α-Cyclodextrin | 89.00 | 65.67 | 24.67 |
| α-D-Glucose | 113.00 | 92.00 | 160.67 |
| D-Melezitose | 2.50 | 0.00 | 7.67 |
| Stachyose | 83.00 | 8.33 | 123.00 |
| α-Ketovaleric Acid | 21.00 | 6.67 | 1.33 |
| Succinic Acid Mono-Methyl Ester | 0.33 | 0.00 | 0.00 |
| Glycyl-L- Glutamine | 3.00 | 22.67 | 0.00 |
| Uridine | 96.00 | 81.33 | 120.33 |
| β-Cyclodextrin | 67.50 | 90.67 | 28.33 |
| α-D-Glucose-1-Phosphate | 10.50 | 8.33 | 0.00 |
| D-Melibiose | 122.50 | 102.33 | 167.67 |
| Sucrose | 123.00 | 91.67 | 158.00 |
| D,L-Lactic Acid | 0.00 | 0.00 | 0.00 |
| m-Tartaric Acid | 0.00 | 0.00 | 0.00 |
| Glycyl-L- Methionine | 6.33 | 18.00 | 0.33 |
| Thymidine-5'-Mono-phosphate | 16.67 | 9.00 | 4.00 |
| Dextrin | 118.00 | 113.67 | 163.67 |
| D-Glucose-6-Phosphate | 4.50 | 6.00 | 2.00 |
| 3-Methyl-D- Glucose | 100.00 | 72.00 | 157.00 |
| D-Trehalose | 6.25 | 1.67 | 8.33 |
| L-Lactic Acid | 0.00 | 0.00 | 1.00 |
| Urocanic Acid | 44.00 | 11.00 | 18.33 |
| Glycyl-L- Proline | 15.33 | 28.33 | 1.67 |
| Uridine-5'- Mono-phosphate | 16.00 | 3.67 | 10.67 |

**Table 2S.** API RAPID ID 32A test result

| **RAPID ID 32A Reactions** | **BC** | **BI** | **163** |
| --- | --- | --- | --- |
| ß-Glucuronidase | - | + | weak |
| Alkaline phosphatase | + | v | + |
| Arginine arylamidase | - | + | + |
| Phenylalanine arylamidase | + | v | - |
| Leucine arylamidase | - | - | weak |
| Tyrosine arylamidase | + | - | + |
| Glycine arylamidase | - | v | + |
| Histidine arylamidase | + | v | + |

**Table 3S.** API 20A test result

| **API 20A Reactions** | **BC** | **BI** | **163** |
| --- | --- | --- | --- |
| melezitose | - | - | weak |
| sorbitol | - | - | weak |

**Table 4S.** Assembly information of MSB 163

| **Assembly information** |  |
| --- | --- |
| Contigs | 3 |
| GC Content | 42.95 |
| Plasmids | 2 |
| Contig L50 | 1 |
| Genome Length | 6,440,948 bp |
| Contig N50 | 6,248,480 |
| Completeness | 99.25% |
| Contamination | 0.12 (very low) |

**Table 5S.** Plasmid one features

| **Feature id 1** | **type** | **location** | **start** | **stop** | **strand** | **function** |
| --- | --- | --- | --- | --- | --- | --- |
| PHJGEONG_05200 | CDS | 2_1+978 | 1 | 978 | + | RepA |
| PHJGEONG_05201 | CDS | 2_992+192 | 992 | 1183 | + | hypothetical protein |
| PHJGEONG_05202 | CDS | 2_1286+522 | 1286 | 1807 | + | ribbon-helix-helix, copG family protein (protein repressor, shortest described to date) |
| PHJGEONG_05203 | CDS | 2_2198-342 | 2198 | 1857 | - | hypothetical protein |
| PHJGEONG_05204 | CDS | 2_2398+174 | 2398 | 2571 | + | Glycosyl transferase, family 2 |
| PHJGEONG_05205 | CDS | 2_2656+978 | 2656 | 3633 | + | Glycosyl transferase, family 2 |
| PHJGEONG_05206 | CDS | 2_3640+1362 | 3640 | 5001 | + | Glycosyl hydrolase 39 |
|  | CDS | 2_5201-174 | 5201 | 5028 | - | Hypothetical protein |
| PHJGEONG_05207 | CDS | 2_6199-876 | 6199 | 5324 | - | mobB (conjugation transfer) |
| PHJGEONG_05208 | CDS | 2_6801-606 | 6801 | 6196 | - | Clindamycin resistence transfer factor btgA |
| PHJGEONG_05209 | CDS | 2_7252+270 | 7252 | 7521 | + | Type II toxin-antitoxin system Phd/YefM family antitoxin |
| PHJGEONG_05210 | CDS | 2_7496+339 | 7496 | 7834 | + | ParE_toxin,RelE |
| PHJGEONG_05211 | CDS | 2_8144-162 | 8144 | 7983 | - | hypothetical protein |

**Table 6S.** Plasmid two features

| **feature_id1** | **type** | **location** | **start** | **stop** | **strand** | **function** |
| --- | --- | --- | --- | --- | --- | --- |
| PHJGEONG_05212 | CDS | 3_1+1131 | 1 | 1131 | + | RepB protein (Rep_3) |
| PHJGEONG_05213 | CDS | 3_1398-231 | 1398 | 1168 | - | charged multivesicular body protein 2b-B |
| PHJGEONG_05214 | CDS | 3_1870-453 | 1870 | 1418 | - | Relaxase/mobilization nuclease domain-containing protein |
| PHJGEONG_05215 | CDS | 3_2621-795 | 2621 | 1827 | - | Relaxase mobilization |
| PHJGEONG_05216 | CDS | 3_2917-300 | 2917 | 2618 | - | relaxase/mobilization nuclease domain-containing protein, partial |
| PHJGEONG_05217 | CDS | 3_3307-198 | 3307 | 3110 | - | relaxase/mobilization nuclease domain-containing protein |
| PHJGEONG_05218 | CDS | 3_3590-273 | 3590 | 3318 | - | Addiction module toxin, Txe/YoeB |
| PHJGEONG_05219 | CDS | 3_3841-252 | 3841 | 3590 | - | PhdYeFM_antitox |
| - | CDS | 3_4013-132 | 4013 | 3882 | - | hypothetical protein |
| - | CDS | 3_4147-138 | 4147 | 4010 | - | type II toxin-antitoxin system YoeB family toxin |

**Table 7S.** Cellular fatty acid contents (%) of MSB163, *B. cellulosilyticus* and *B. intestinalis* determined by Microbial ID by Fatty Acid Methyl Esters (FAME) Analysis. Values are percentages of total fatty acid detected using TSBA6 library and ANAERO6 calculation method. The highest values are highlighted in orange. Summed features combine fatty acids eluting at the same retention time.

| **ANAER6** | **MSB163** | ***B. cellulosilyticus*** | ***B. intestinalis*** |
| --- | --- | --- | --- |
| C 13:0 ISO FAME | 0.32 | 0.14 | 0.35 |
| C13:0 ANTEISO FAME | 0.38 | 0.13 | 0.18 |
| Sum in feature 1 | 1.22 | 0.86 | 1.08 |
| Sum in feature 3 | 1.66 | 2.76 | 4.1 |
| C14:0 ISO FAME | 1.17 | 0.49 | 0.47 |
| C14:0 FAME | 2.72 | 0.84 | 1.45 |
| C15:0 ISO FAME | 5.71 | 3.85 | 7.11 |
| C15:0 ANTEISO FAME | 38.04 | 35.73 | 30.53 |
| C15:0 FAME | 2.73 | 2.75 | 1.95 |
| C15: ISO DMA |  | 0.17 |  |
| C:15 ANTEISO DMA |  | 0.38 |  |
| Sum in feature 5 |  | 0.28 | 0.28 |
| C16:0 - ISO FAME | 0.18 | 0.17 | 0.12 |
| C16:1 CIS 9 FAME | 0.35 | 0.22 | 0.37 |
| C16:0 FAME | 14.05 | 10.77 | 12.13 |
| C15:0 ISO 3OH FAME | 0.24 | 0.15 | 0.35 |
| Sum in feature 6 |  |  | 0.08 |
| C15:0 3OH FAME | 1.1 | 1.1 | 0.86 |
| C:17:0 ANTEISO FAME | 0.18 | 0.2 | 0.18 |
| Sum in feature 8 |  |  | 0.09 |
| C:10 ISO FAME |  | 0.12 |  |
| Sum in feature 9 | 1.08 | 1.2 | 0.89 |
| C16:0 3OH FAME | 9.5 | 8.98 | 9.63 |
| C18:2 CIS 9,12 FAME | 3.52 | 3.3 | 2.84 |
| C18:1 CIS 9 FAME | 1.98 | 1.8 | 3.41 |
| C18:1 T 11 ?? FAME |  |  | 0.19 |
| C18:0 FAME | 0.87 | 0.63 | 0.84 |
| Sum in feature 11 | 10.11 | 17.24 | 17.45 |
| C17:0 ANTE 3OH FAME | 2.9 | 5.29 | 2.72 |
| C17:0 3OH ? FAME |  | 0.43 | 0.21 |
| Summed feature 1 | 1.92 | 0.86 | 1.08 |
| Summed feature 3 | 1.66 | 2.76 | 4.1 |
| Summed feature 5 |  |  | 0.28 |
| Summed feature 6 |  |  | 0.08 |
| Summed feature 8 |  |  | 0.09 |
| Summed feature 9 | 1.08 | 1.2 | 0.89 |
| Summed feature 11 | 10.11 | 17.24 | 17.45 |

**Table 8S.** Cellular fatty acid contents (%) of MSB163, *B. cellulosilyticus* and *B. intestinalis*. Values are percentages of total fatty acid detected using TSBA6 library and Clin6 calculation method. The highest values are highlighted in orange. Summed features combine fatty acids eluting at the same retention time.

| **Clin6** | **MSB163** | ***B. cellulosilyticus*** | ***B. intestinalis*** |
| --- | --- | --- | --- |
| C13:0 ISO | 0.33 | 0.15 | 0.37 |
| 13:0 anteiso | 0.39 | 0.14 | 0.19 |
| 13:1 at 12-13 | 1.27 | 0.91 | 1.15 |
| unknown 13.565 |  |  |  |
| 14:0 iso | 1.23 | 0.51 | 0.5 |
| 14:00 | 2.84 | 0.89 | 1.54 |
| 15:0 iso | 5.97 | 4.07 | 7.56 |
| 15:0 anteiso | 39.8 | 37.72 | 32.44 |
| 15:00 |  |  |  |
| Sum in feature 2 |  |  | 0.29 |
| 16:0 iso | 0.19 | 0.18 | 0.12 |
| C14:0 2OH |  | 0.4 |  |
| Sum in feature 2 |  | 0.29 |  |
| C10:0 ISO |  | 0.18 |  |
| Sum In Feature 3 | 0.37 | 0.24 | 0.39 |
| C16:0 | 14.7 | 11.37 | 12.89 |
| 15:0 iso 3OH | 0.25 | 0.16 | 0.37 |
| 15:0 3OH | 1.15 | 1.16 | 0.08 |
| unknown 16.582 |  |  |  |
| 17:0 anteiso | 0.19 | 0.22 | 0.19 |
| Sum in feature 3 | 0.37 |  |  |
| C17:0 |  | 0.12 | 0.17 |
| C17:0 w8c |  |  | 0.1 |
| 16:0 iso 3OH | 1.13 | 1.27 | 0.95 |
| 16:0 3OH | 9.94 | 9.48 | 10.22 |
| Sum In Feature 5 | 3.68 | 3.48 | 3.02 |
| 18:1 w9c | 2.07 | 1.9 | 3.62 |
| sum in feature 8 |  |  | 0.2 |
| 18:00 | 0.9 | 0.67 | 0.89 |
| 17:0 iso 3OH | 10.57 | 18.18 | 18.52 |
| 17:0 2OH | 3.03 | 5.58 | 2.89 |
| 17:0 3OH |  | 0.45 | 0.23 |
| 20:4 w6,9,12,15c |  | 0.29 | 0.21 |
|  |  |  |  |
| Summed feature 2 |  | 0.29 | 0.29 |
| Summed Feature 3 | 0.37 | 0.24 | 0.39 |
| Summed Feature 5 | 3.68 | 3.48 | 3.02 |
| Summed feature 8 |  |  | 0.2 |

**FIGURES**

**
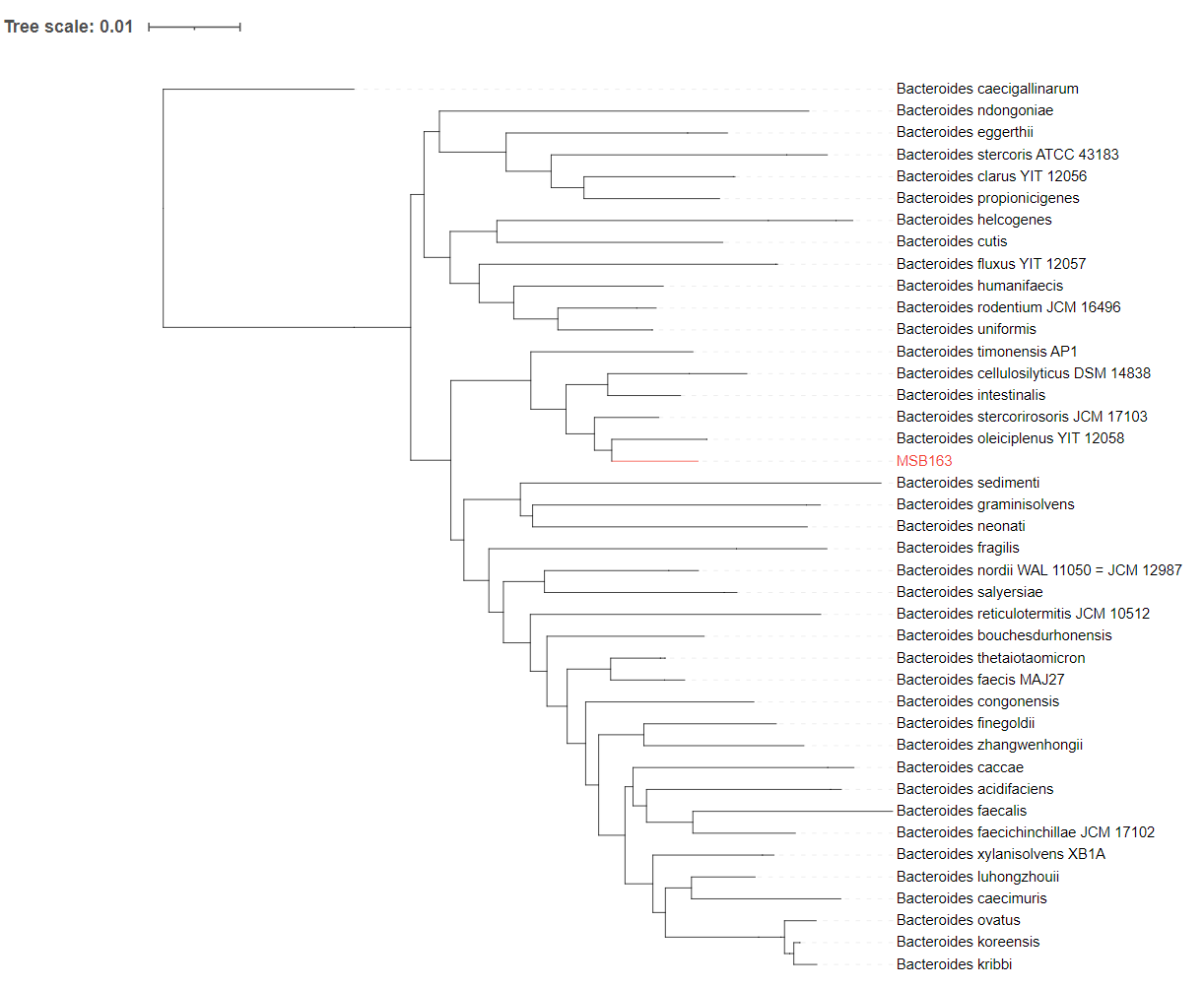
**

**Figure 1S.** Phylogenetic tree based on 16S rRNA gene, including strain MSB163 and other *Bacteroides* assemblies available on NCBI.

**
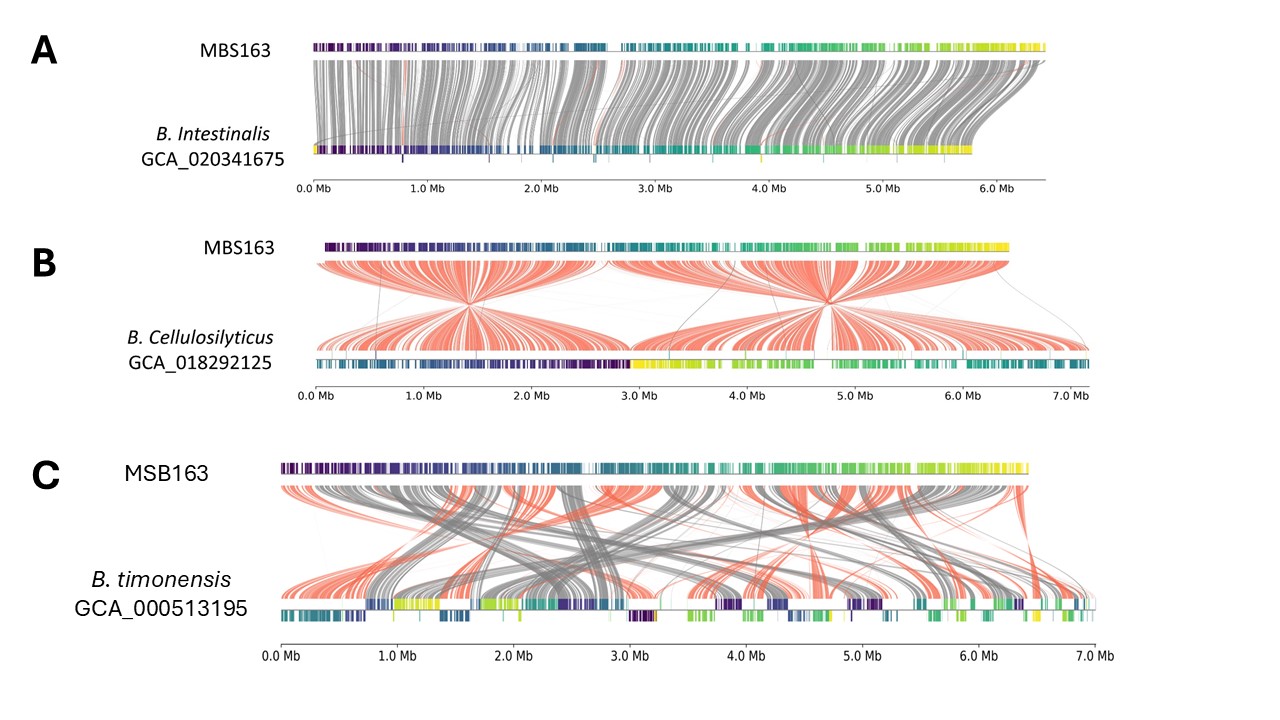
**

**Figure 2S.** Synteny plot of MSB163 and its closely related species. a) Synteny plot of MSB163 and *B. intestinalis*, b) Synteny plot of MSB163 and *B. cellulosilyticus*, c) Synteny plot of MSB163 and *B. timonensis*.


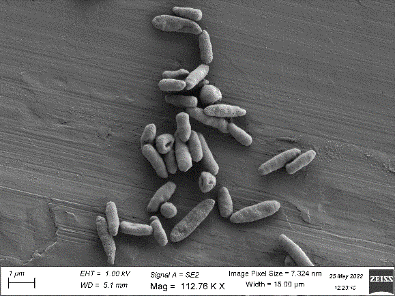

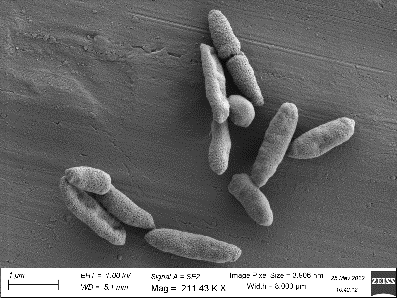


**Figure 3S.** Scanning Electron Microscopy (SEM) images of MSB163.


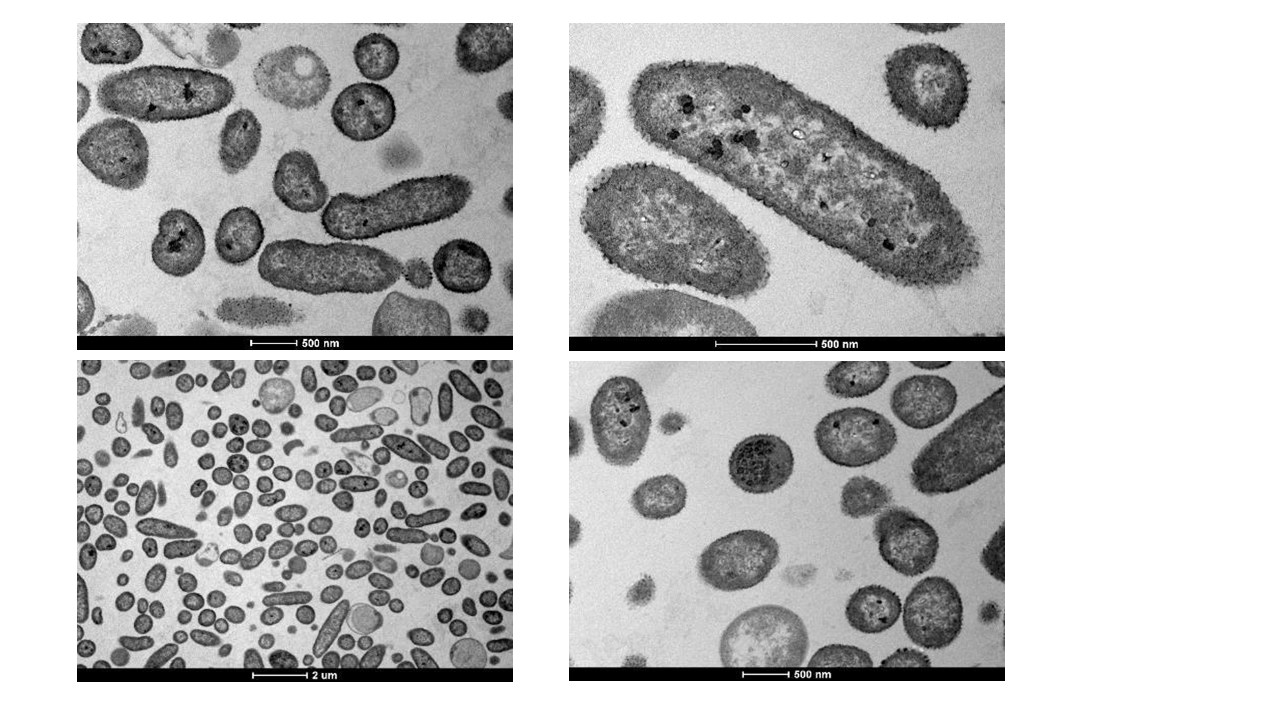


**Figure 4S.** Transmission Electron Microscopy (TEM) images of MSB163


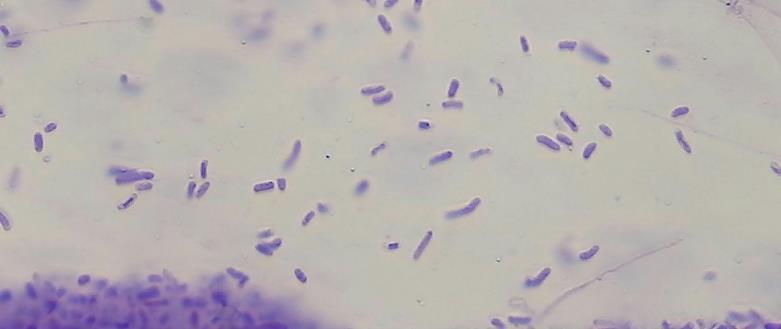


**Figure 5S.** MSB163 seen under the microscope.

**Figure 6S**. Growth of MSB163, *B. cellulosilyticus* and *B. intestinalis* at different temperatures measured after 24 hours. The experiment was done in triplicate and the standard deviation is shown in the graph.

**Figure 7S.** Growth of MSB163, *B. cellulosilyticus* and *B. intestinalis* at different NaCl concentration. The experiment was done in triplicate and growth was measured after 24 hours. The standard deviation is shown on the graph.

**Figure 8S.** Growth of strain MSB163 in different media and carbohydrate sources.
